# Acupuncture ameliorates neurological function in rats with cerebral ischemia‐reperfusion by regulating the opening of large‐conductance Ca^2+^‐activated potassium channels

**DOI:** 10.1002/brb3.2286

**Published:** 2021-08-01

**Authors:** Lin Han, Yong Wang, Guanran Wang, Yingying Chen, Haiping Lin, Yanan Zhang, Yan Shen

**Affiliations:** ^1^ First Teaching Hospital of Tianjin University of Traditional Chinese Medicine Tianjin China; ^2^ National Clinical Research Center for Chinese Medicine Acupuncture and Moxibustion Tianjin China; ^3^ Xiamen Hospital of Traditional Chinese Medicine Xiamen China; ^4^ Tianjin University of Traditional Chinese Medicine Tianjin China; ^5^ Tianjin Key Laboratory of Acupuncture and Moxibustion Tianjin China

**Keywords:** acupuncture, cellular electrophysiology, cerebral ischemia‐reperfusion, GV26, large‐conductance Ca^2+^‐activated potassium channels (BKCa), patch‐clamp

## Abstract

Acupuncture has a good effect on improving neurological function after cerebral ischemia‐reperfusion, but there are few studies on the neuroprotective effect of acupuncture from the perspective of ion channel cellular electrophysiology. Studies have shown that the over activation of large‐conductance Ca^2+^‐activated potassium channel （BKCa） after cerebral ischemia‐reperfusion can reduce the excitability of neurons and induce apoptosis. This study intends to establish middle cerebral artery occlusion/reperfusion (MCAO/R) model, with acupuncture at GV26 as the intervention measure, using patch‐clamp technique to record the electrophysiological changes of BKCa channel. The results showed that the neurological function score of MCAO/R rats was significantly decreased, and the conductance, open dwell time and open probability of BKCa channel in hippocampal CA1 neurons of MCAO/R rats were significantly increased. Acupuncture at GV26 could significantly improve the neurological function scores of MCAO/R rats, and reduce the conductance, open dwell time, and open probability of BKCa channel. The effect of acupuncture at GV26 was significantly better than acupuncture at non‐acupuncture point. The neuroprotective effect of acupuncture at GV26 after cerebral ischemia‐reperfusion may be related to regulating the electrophysiological characteristics of BKCa channel opening.

## Graphical abstract

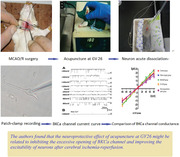

## 1 INTRODUCTION

Stroke is one of the most common diseases in the world endangering human life and health, and 87% of stroke patients are ischemic stroke caused by vascular occlusion (Powers et al., 2018). The mortality and disability rate of ischemic stroke is high, which greatly affects the living ability and quality of patients (Chen et al., 2020; Poustchi et al., 2021). The recovery of blood flow within a certain period of time after cerebral ischemia will aggravate tissue damage and dysfunction, and even lead to irreversible damage, which is called cerebral ischemia‐reperfusion injury (Pan et al., 2007). The main mechanism underlying cerebral ischemia‐reperfusion injury is excitotoxicity and calcium overload, which causes a series of cascade reactions that lead to delayed neuronal necrosis (Lo et al., 2003).

After cerebral ischemia‐reperfusion injury, large‐conductance Ca^2+^‐activated potassium channels (BKCa) are excessively activated and expressed, resulting in excessive K^+^ efflux and hyperpolarization of cell membrane (Hu et al., 2002; Wang et al., 2016). It is hypothesized that the activation of BKCa channel mediates hypoxia/reoxygenation (H/R) and ischemia/reperfusion (I/R) induced neuronal apoptosis (Chen et al., 2013). In vitro intracellular recording study shows the enhanced activity of BKCa channels in hippocampal CA1 pyramidal neurons after ischemia may partially contribute to the post‐ischemic decrease in neuronal excitability and increase in fast afterhyperpolarization (FAHP) (Gong et al., 2000). It is possible that persistent hyperactivity of BKCa channels may not only cause progressive depression of neuronal excitability but also trigger the process of delayed neuronal cell death (Gong et al., 2002).

Acupuncture has a definite therapeutic effect on cerebral ischemia‐reperfusion injury. At present, the study of the pathogenesis of cerebral ischemia‐reperfusion mainly concentrates on neuronal apoptosis (Liu et al., 2018). Studies have shown that acupuncture can prevent apoptosis after cerebral ischemia‐reperfusion by regulating autophagy, reducing inflammatory response, inhibiting excitotoxicity and calcium overload, antioxidant stress, and other ways (Guo et al., 2015; Jiang et al., 2019; Wang et al., 2017; Wang et al., 2017). Cells constitute the basic units of living organisms, and the synergy between cells facilitates the various functions of the body. However, few studies have focused on the neuroprotective effect of acupuncture from the perspective of cellular electrophysiology in vitro. Patch‐clamp technique is a well‐developed cellular electrophysiological method. Using patch‐clamp technology, the electrical changes of cell membrane ion channels can be observed to elucidate the cellular electrophysiological mechanisms of acupuncture (Zhang, 2012).

In this study, patch‐clamp technique was used to observe the effect of acupuncture at GV26 on cellular electrophysiological characteristics of BKCa channel in hippocampal CA1 neurons after focal cerebral ischemia‐reperfusion in rats, and to explore whether acupuncture can play a role in brain protection by regulating the opening of BKCa channels and affecting neuronal excitability.

## 2 METHODS

### 2.1 Animals and groups

A total of 50 healthy adult male Sprague‐Dawley rats (230 ± 10 g of body weight), were purchased from Beijing Vital River Laboratory Animal Technology Co. Ltd. Experimental animal certificate number: SCXK (Beijing) 2016‐0011. All animals were housed in a conditioned environment of controlled temperature (20～25°C), on an illumination schedule of 12 h light/12 h dark and fed a standard pellet diet, with sterile drinking water provided ad libitum. Rats were randomly divided into five groups as follow: the control group, the sham surgery group, the model group, the GV26 group, and the non‐acupuncture point group, 10 rats in each group. All experiments were performed according to the National Institute of Health Guide for the Care and Use of Laboratory Animals (NIH Publications No.80‐23) and were approved by the Animal Ethics Committee, Tianjin University of Traditional Chinese Medicine in China (No. TCM‐LAEC2014008).

### 2.2 Acupuncture treatment

**FIGURE 1:**
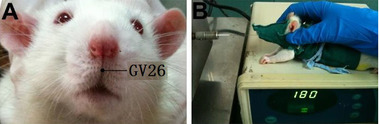
A: Location of GV 26 in rats. B: Manipulation of lifting and inserting at GV 26

The location of GV26 was on the face, at the junction of the superior 1/3 and the middle 1/3 of the philtrum in clinical application. Referencing *Experimental Acupuncture Science*, we took the location of cleft lip 1 mm below the nasal as the equivalent acupuncture point of GV26 and the point 1.5 cm lateral to the left side of GV26 as the non‐acupuncture point (Guo, 2016). At present, in acupuncture research, non‐acupuncture points are often selected beside the acupuncture points as control (Liu & Cai, 2010). Acupuncture needles were 40 mm in length and 0.3 mm in diameter (*Huatuo*, Suzhou medical supplies factory, China). The needle was obliquely inserted toward the nasal septum at a depth of 2 mm for GV26 and vertically inserted at a depth of 2 mm for the non‐acupuncture point. GV26 and the non‐acupuncture point were both treated with lifting and inserting acupuncture manipulation at a frequency of 2 times per second, the operation time of acupuncture was 60 s, once every 12 h for a total of six times within 72 h. In order to ensure the controllability and stability of acupuncture frequency, depth, and operation time, and avoid manual operation errors, the acupuncture lifting and inserting operation was completed by acupuncture manipulator developed by Chongqing Haifu Medical Technology Co., Ltd instead of manual operation (Figure 1).

### 2.3 Middle cerebral artery occlusion/reperfusion surgery

Middle cerebral artery occlusion/reperfusion (MCAO/R) was performed as previously described (Longa et al., 1989). Rats were anesthetized with pentobarbital (50 mg kg^−1^, i.p.) and were fixed in supine position on the surgical table. The left common carotid artery (CCA), internal carotid artery, and external carotid artery (ECA) were surgically exposed via a midline incision and carefully dissected from the surrounding tissue. After CCA was clamped and ECA was ligatured by 0^#^ suture, a nylon filament (0.26 mm in diameter) with round tip made by heating near a flame was inserted intraluminally into the ICA about 18 to 20 mm until a slight resistance was felt (which means the tip of the filament reached the origin of middle cerebral artery) and ligatured. Then, the clamp in CCA was released and the incision was sutured. After blocking the blood supply for 2 h, the nylon filament was removed to achieve reperfusion. Sham surgery group received the same operation but without nylon filament insertion. The rats’ rectum temperatures were maintained at 37 ± 1°C during the surgery by an electric blanket. Neurological function of all awake animals was evaluated following MCAO/R surgery by using Zausinger 6‐point neurological function scores (Zausinger et al., 2000). Rats with neurological function scores ranging from grade 1–3 were qualified as an established MCAO/R model and entered the next phase of the experiment. 30 rats were used in MCAO/R surgery. No animal died during the surgery. 3 rats were eliminated due to the unqualified neurological function scores. In the follow‐up experiment, the animal models were supplemented to ensure that each group had 10 qualified MCAO/R models. The success rate of MCAO/R surgery was 90% in this experiment.

### 2.4 Neurological evaluation

Neurological deficits were assessed after MCAO/R surgery and 3 days after acupuncture treatment by professional staff blinded to the surgery and acupuncture treatment. The scale was carried out as follows: (0), without spontaneous activity; (1), falling to the contralateral side; (2), severe circling when tail pull; (3), lowered resistant to contralateral push; (4), unable to extend the contralateral forelimb; (5), no deficit. The lower the score, the more serious the neurological impairment.

### 2.5 Hippocampal HE staining

The hippocampal tissue of ischemic side was dissected and fixed with 10% formalin. The tissue was dehydrated with ethanol and xylene, soaked in wax, embedded, and sliced. The nucleus was stained with hematoxylin and the cytoplasm was stained with eosin. Then the tissue was dehydrated gradiently and sealed with neutral gum. The images were collected and analyzed by microscope.

### 2.6 Neuron acute dissociation procedures

The following solutions (mmol L^−1^) were used during the course of the dissociation procedures. Deionized water was used for preparing various kinds of solutions and all the solutions were filtered by a 200 mesh filter before use to make sure the liquid is clean enough.

HSS, Hyper osmotic sucrose solution: Sucrose 234, KCl 2.5, Na_2_HPO_4_ 1, CaCl_2_•2H_2_O 0.1, HEPES 15, glucose 11, MgSO_4_•7H_2_O 4, PH 7.3 with 1 mol L^−1^ NaOH, 300～305 mOsm L^−1^. HSSB, Hvdroxyethyl sulfunic sodium buffer: Hvdroxyethyl sulfunic sodium 132, KCl 2, CaCl_2_•2H_2_O 0.1, MgCl_2_•6H_2_O 4, HEPES 15, glucose 23, PH7.3 with 1 mol L^−1^ NaOH, 300～305 mOsm L^−1^. EBSS, Earle's balanced salt solution: NaHCO_3_ 26, CaCl_2_•2H_2_O 1.8, MgSO_4_ 0.8, KCl 5.35, NaCl 116, NaH_2_PO_4_ 1, glucose 5.56, phenolsulfonphthalein 0.003, bubbled with 95% O_2_+5% CO_2_ for at least 1 h, PH 7.3 with 1 mol L^−1^ NaOH, 300～305 mOsm L^−1^. HBSS, Hanks balanced salt solution: KCl 5.37, KH_2_PO4 0.441, NaCl 137, Na_2_HPO4 0.34, HEPES 11, phenolsulfonphthalein 0.003, MgCl_2_•6H_2_O 4, glucose 5.56, CaCl_2_ 1, PH7.3 with 1 mol L^−1^ NaOH, 300～305 mOsm L^−1^. Bath solution, potassium gluconate 140, NaCl 10, HEPES 10, PH7.3 with 1 mol L^−1^ NaOH, 300～305 mOsm L^−1^.

The rats were terminally anaesthetized with sodium pentobarbital (80 mg kg^−1^, i.p.) and then quickly decapitated. The brain tissue was removed, iced in 0–4°C HSS with 100% O_2_ for 2 min and cut into 400 μm thick slices by vibration slicer (World Precision Instrumellts MA752‐045). The brain slices were rinsed with HSSB for three times and then incubated in EBSS bubbled with 95% O_2_ and 5% CO_2_ at 33°C for 1.5 h. Then, the brain slices were transferred into HSSB, the CA1 area of hippocampus was dissected under the anatomical microscope and was put into HBSS containing the protease XIV (1.3 g L^−1^, Sigma) oxygenated with 100%O_2,_ the enzyme digestion was carried out at 33°C for 30 min. After enzyme digestion, the brain tissue was rinsed in HSSB for three times and then triturated mechanically with a graded series of fire polished Pasteur pipettes to disperse the tissue (the caliber of tip of Pasteur pipettes was 500, 300, and 150 μm, respectively). The cell suspension was then transferred onto a cleaned cover glass and was rinsed with normal bath solution twice after the cell adhered to the cover glass. Then the cover glass was plated onto a dish mounted on the stage of a microscope (Olympus IX71) and the extracellular bath solution was added into the dish. Pyramidal cells in hippocampal CA1 region with uniformly bright appearance and good adhesion to cover glass were selected for patch‐clamp record (Li et al., 2004).

### 2.7 Single channel patch‐clamp recording technique

Patch‐clamp recording pipettes were pulled from glass capillary tubes using a micropipette puller (P‐97, Sutter Instruments). The tip diameter of the pipettes was 0.5～1 μm. The pipette resistance was 6–9 MΩ and the seal resistance could be in excess of 5 GΩ. Recordings were obtained according to the standard patch‐clamp method using an Axon patch 200B amplifier (Axon Instruments) interfaced to a personal computer (Hamill et al., 1981). Voltage commands were generated, and current responses were recorded and analyzed using a computerized acquisition and storage system (PCLAMP, Axon Instruments). Current responses were low‐pass filtered at l kHz and digitized at 5 kHz. Voltage pulses were delivered at 5 s intervals. Electrophysiological recordings were performed at room temperature (2l～24°C).

To determine distributions for channel amplitudes, and open times, a 50% threshold criterion was used to determine the durations of open events. The current amplitudes were determined from Gaussian fits to amplitude histograms from individual patches. Logarithmic distributions of open and closed durations were exponentially fitted with the use of the least‐square algorithm method. Channel open probability (Po) in response to a particular stimulus was calculated by evaluating all applicable sweeps during the entire recording. The total open time during the analyzed portion of the sweep was divided by the analysis time period. The recording pipette solution contained 140 mmol L^−1^ potassium gluconate, 10 mmol L^−1^ NaCl, 10 mmol L^−1^ HEPES, PH 7.3 with 1 mol L^−1^ NaOH. The osmolarity of all recording solutions was adjusted to 320～325 mosmol L^−1^ as necessary. Inside‐out patch‐clamp configuration was used to record BKCa channel current. Holding potential was −80 mV, commanding potential was set from −80 to +80 mV, stepped by 20 mV.

### 2.8 Statistical analysis

All data are expressed as mean ± SD. SPSS 19.0 software (SPSS, Chicago, IL, USA) was used for the data analysis. Kolmogorov–Smirnov test was used to examine data distribution. The measurement data was compared using one‐way ANOVA or Kruskal–Wallis test according to data distribution. *P *< 0.05 level was considered statistically significant.

## 3 RESULTS

### 3.1 Analysis of experimental animal number

10 rats died within 72 h after MCAO/R surgery, including 4 rats in model group, 3 rats in GV26 group, 3 rats in non‐acupuncture point group, and an additional 10 qualified animal models were added to ensure 10 MCAO/R models for each group.

### 3.2 Histomorphological characteristics of hippocampus in each group

**FIGURE 2:**
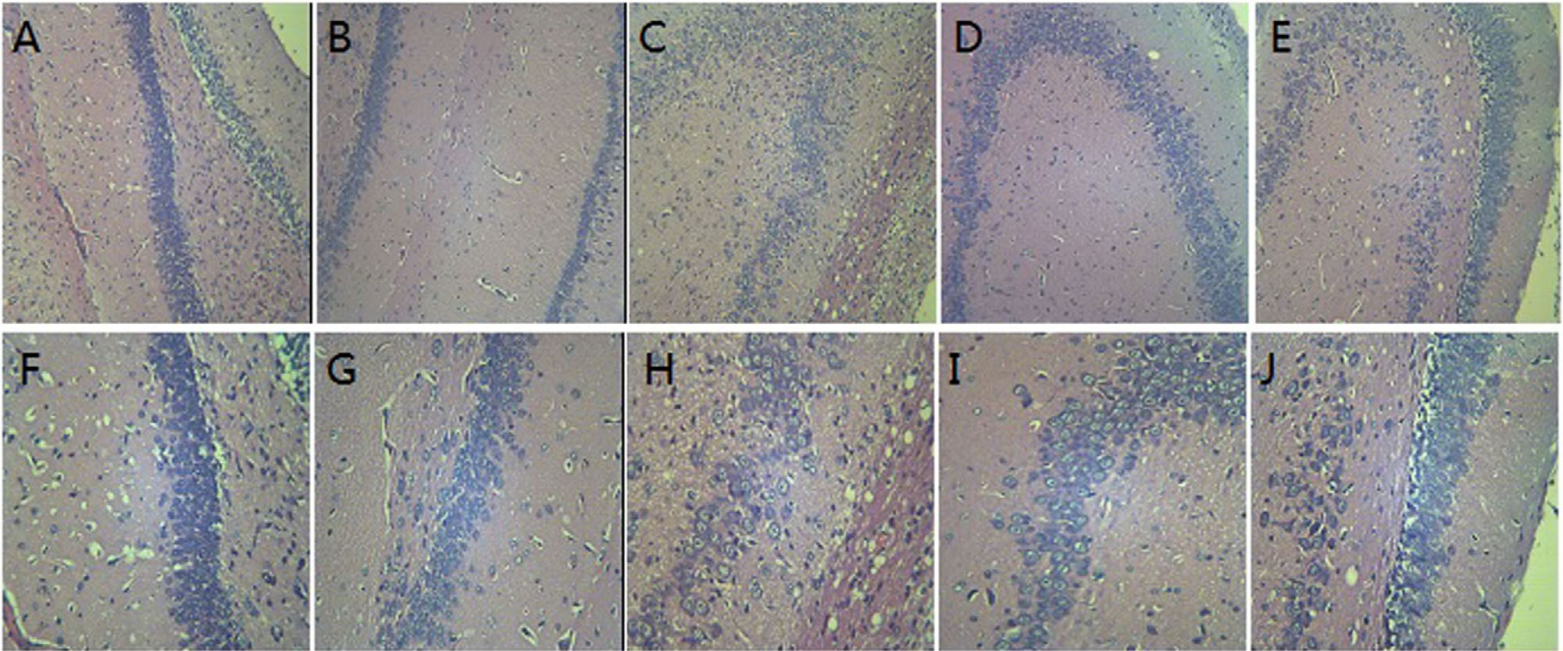
Histological changes of hippocampal CA1 in each group of rats ( *n* = 5) A, F: Normal group B, G: Sham surgery group; C, H: MCAO/R group; D, I: GV26 group; E, F: Non‐acupuncture point group (A, B, C, D, E: HE staining × 100; F, G, H, I, J: HE staining × 400)

In normal group and sham surgery group, hippocampal neurons and glial cells were arranged orderly, the cytoplasm was evenly stained, and the structure was normal. In model group, hippocampal neurons were arranged in disorder, the cell morphology was incomplete and swollen, a large number of neurons were lost and necrotic, the number of cells decreased significantly, and some nuclei were karyolytic and pyknotic. In EA group, the degree of cell disorder in the hippocampus was alleviated, and the number of degenerative and necrotic nerve cells significantly decreased. In non‐acupuncture point group, the number of hippocampal neurons decreased, some neurons were swollen and necrotic, and some nuclei showed the pathological characteristics of karyolysis and pyknosis (Figure 2).

### 3.3 Acupuncture improved neurological function scores after cerebral ischemia/reperfusion in rats

**FIGURE 3:**
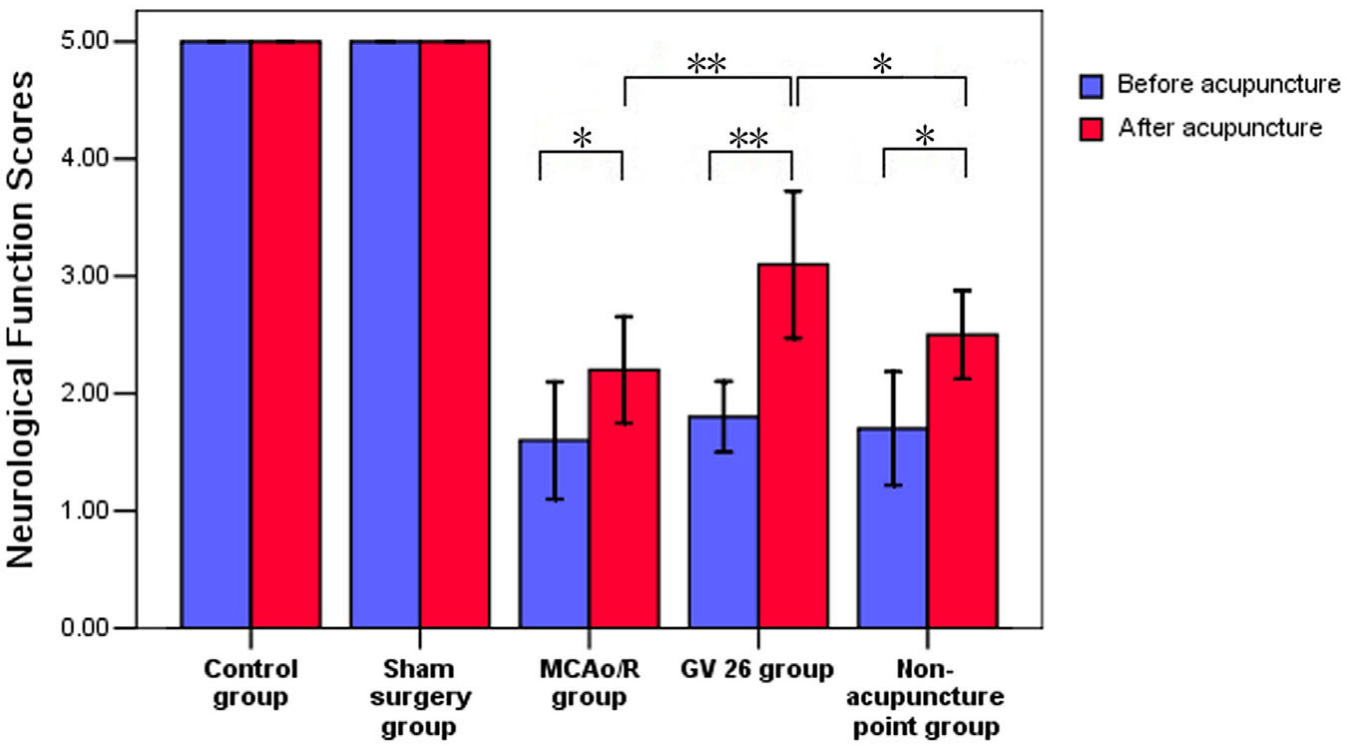
Comparison of neurological function scores in each group (*n* = 10)

The result of neurological function scores conformed to normal distribution with homogeneous variance. Differences among groups were examined using AVONA followed by LSD for post hoc multiple comparisons. There was no significant difference between model group, GV26 group and non‐acupuncture point group with regard to neurological function scores prior to treatment (*P *> 0.05). Compared with that before treatment, the neurological function scores in model group, GV26 group, and non‐acupuncture point group were all significantly increased after treatment (*P *< 0.05, *P *< 0.01). Compared with the sham surgery group at the same time, the neurological function scores of the model group decreased significantly before and after treatment (*P *< 0.01). Compared with the model group and non‐acupuncture point group, the neurological function scores of GV26 group increased more significantly after treatment (*P *< 0.05, *P *< 0.01) (Table 1, Figure 3).

**TABLE 1:** Neurological function scores before and after acupuncture treatment (*X* ± *s*)

		**Neurological function scores**	
**Groups**	**Number (*n*)**	**Before treatment**	**After treatment**
Control group	10	5.00 ± 0.00	5.00 ± 0.00
Sham surgery group	10	5.00 ± 0.00	5.00 ± 0.00
Model group	10	1.60 ± 0.70^Δ^	2.20 ± 0.63*, ^Δ^
GV26 group	10	1.80 ± 0.42	3.10 ± 0.88**^▲°^
Non‐acupuncture point group	10	1.70 ± 0.68	2.50 ± 0.53*

* *P *< 0.05.

** *P *< 0.01 versus before treatment; Δ*P *< 0.01 versus sham surgery group; ▲*P *< 0.01 versus model group; °*P *< 0.05 versus non‐acupuncture point group.

### 3.4 Electrophysiological properties of BKCa channels

**FIGURE 4:**
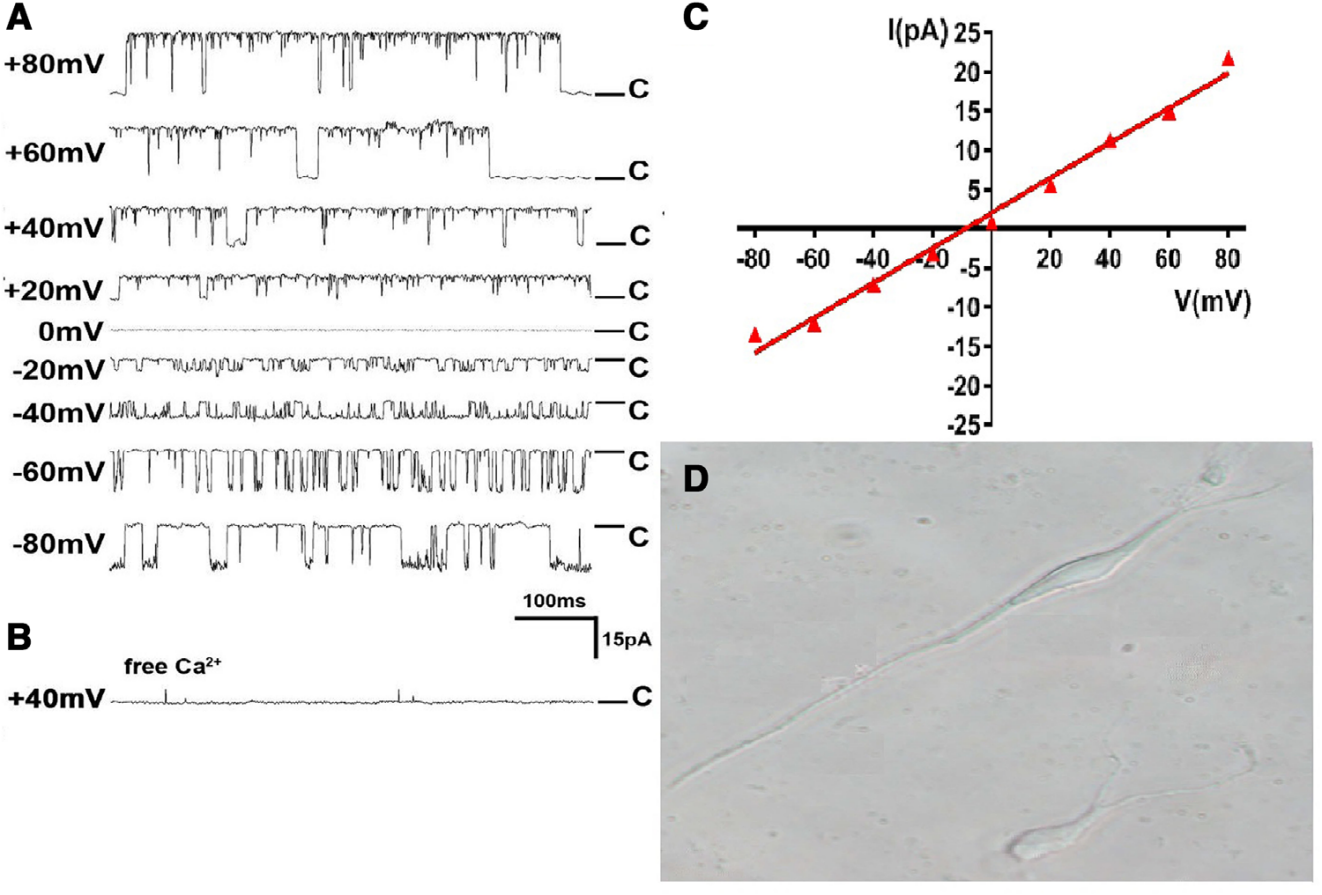
A) BKCa channel current curve of hippocampal CA1 pyramidal neurons in control group (concentration of free Ca^2+^ in extracellular bath solution was 1000 nmol L^−1^ and concentration of K^+^ was 140 mmol L^−1^ both in extracellular bath solution and recording pipette solution). B) BKCa channel current curve when command voltage was +40 mV and there was no free Ca^2 +^ in extracellular bath solution. C) *I*–*V* curve of BKCa channel in hippocampal CA1 pyramidal neurons in control group (*n* = 5). D) Pyramidal neurons in hippocampal CA1 area in healthy rats

The somas of healthy hippocampal neurons obtained after enzyme digestion and mechanical trituration had a uniform bright field of vision, which was in the shape of pyramidal or fusiform, without mottled dark patches. The proximal dendrite structure remains, and the apical dendrite was maintained. BKCa channels possess high sensitivity to intracellular calcium, as well as, to changes in membrane voltage. In the symmetrical 140 mmol L^−1^ K^+^ solution (extracellular bath solution was equivalent in the concentration of K^+^ to recording pipette solution), BKCa channels were mostly open in clusters and bursts like, the current amplitude increased with the process of hyperpolarization or depolarization, reflecting the voltage dependent characteristics of BKCa channels. Under the same clamping potential, the current amplitude of BKCa channel is basically the same, and the waveform is rectangular square wave with different wave width. In the absence of free Ca^2+^ in the extracellular bath solution, the BKCa channel was basically not open, showing calcium dependent characteristics. The slope conductance of the BKCa channel was 223 ± 27.3 pS, which was fitted by the linear regression equation from the current‐voltage relation curve (*I*–*V* curve, patches *n* = 5, Figure 4).

### 3.5 Acupuncture reduces the conductance of BKCa channel in hippocampal CA1 neurons after cerebral ischemia/reperfusion in rats

**FIGURE 5:**
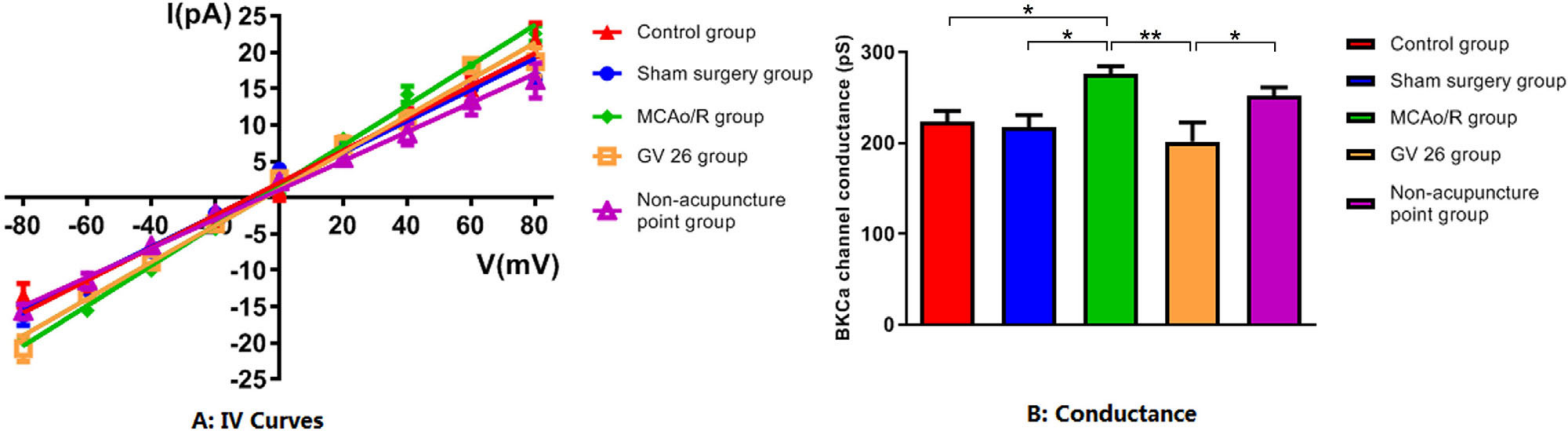
Comparison of BKCa channel conductance of hippocampal CA1 neurons in each group (*n* = 5)

The result of conductance of BKCa channel conformed to normal distribution with homogeneous variance. Differences among groups were examined using AVONA followed by LSD for post hoc multiple comparisons. Compared with the control group, there was no significant difference of BKCa channel conductance in sham surgery group (*P *> 0.05). Compared with the control group and sham surgery group, the BKCa channel conductance in model group increased significantly (*P *< 0.05). Compared with the model group and the non‐acupuncture point group, the BKCa channel conductance in GV26 group decreased significantly (*P *< 0.05, *P *< 0.01) (Table 2, Figure 5).

**TABLE 2:** Comparison of conductance of BKCa channel in hippocampal CA1 neurons of each group (patches *n* = 5)

**Group**	**Patches (*n*)**	**Conductance pS**
Control group	5	223.00 ± 27.34
Sham surgery group	5	217.6 ± 29.59
Model group	5	276.1 ± 18.56* ^Δ^
GV26 group	5	201.4 ± 51.54^▲°^
Non‐acupuncture point group	5	252.0 ± 20.30

* *P *< 0.05, versus control group; Δ*P *< 0.05, versus sham surgery group; ▲*P *< 0.01 versus model group; °*P *< 0.05 versus non‐acupuncture point group.

### 3.6 Acupuncture reduces the open probability and open dwell time of BKCa channel in hippocampal CA1 neurons after cerebral ischemia/reperfusion in rats

**FIGURE 6:**
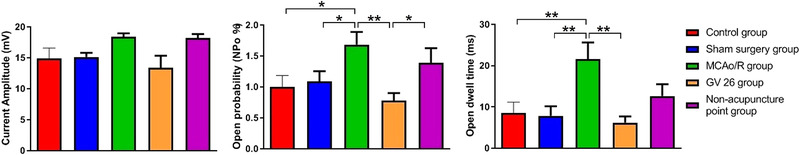
Comparison of current amplitude, open probability, and open dwell time in each group (*n* = 6)

In each group, 6 successfully sealed patches were randomly selected for patch‐clamp experimental data analysis. As the current curve recorded under +60 mV clamping voltage has a stable baseline, relatively less noise and stable channel opening, the current curve recorded under +60 mV clamping voltage is fitted and analyzed in the experiment. The results of open probability and open dwell time conformed to normal distribution with homogeneous variance. Differences among groups were examined using AVONA followed by LSD for post hoc multiple comparisons. The results of current amplitude conformed to the normal distribution yet with heterogenous variance. Tamhane's T2 test was used to compare between experimental groups. Compared with the control group, there was no difference of the current amplitude, open probability, and open dwell time in sham surgery group (*P *> 0.05); compared with the control group and sham surgery group, the open probability and open dwell time of the model group were significantly increased (*P *< 0.05, *P *< 0.01), the current amplitude showed an increasing trend, but there was no statistical significance (*P *> 0.05); compared with the model group, the open probability and open dwell time of GV26 group were significantly decreased (*P *< 0.01), the current amplitude showed a decreasing trend without statistical significance (*P *> 0.05); compared with non‐acupuncture point group, the open probability of GV26 group decreased significantly (*P *< 0.05), the current amplitude and open dwell time showed an decreasing trend, but there was no statistical significance (*P *> 0.05) (Table 3, Figure 6).

**TABLE 3:** Comparison of current amplitude, open probability and open dwell time of BKCa channel in hippocampal CA1 neurons of each group (patches *n* = 6)

**Group**	**Patches**	**Current amplitude mV**	**Open probability ％**	**Open dwell time ms**
Control group	6	14.86 ± 3.85	1.00 ± 0.45	8.57 ± 6.31
Sham surgery group	6	14.37 ± 2.31	1.09 ± 0.40	7.37 ± 6.20
Model group	6	18.38 ± 1.27	1.68 ± 0.50*, ^Δ^	21.62 ± 9.86**^ΔΔ^
GV26 group	6	13.36 ± 4.82	0.78 ± 0.30^▲°^	6.20 ± 3.72^▲^
Non‐acupuncture point group	6	18.19 ± 1.46	1.39 ± 0.59	14.95 ± 10.00

* *P *< 0.05.

** *P *< 0.01 versus control group; ^Δ^ *P *< 0.05, ^ΔΔ^ *P *< 0.01 versus sham surgery group; ^▲^ *P *< 0.01 versus model group; °*P *< 0.05 versus non‐acupuncture point group.

## 4 DISCUSSION

Shuigou (GV26) is the acupoint belonging to the Governor vessel and it is the main point of "*Xingnao Kaiqiao*" acupuncture method, which is a series of systematic acupuncture methods with yin channels in domination, assisted by specified standards of quantity manipulation and is effective in treating stroke (Yang et al., 2015). Meanwhile, GV26 is the intersection point of the Large Intestine Channel of Hand‐Yangming, the Stomach Channel of Foot‐Yangming and the Governor vessel. Acupuncture at GV26 can regulate the governor vessel, calm the mind, restore consciousness and induce resuscitation. Studies have confirmed that acupuncture at GV26 plays an important protective role in cerebral ischemic injury. Fan et al (Fan et al., 2009) found that acupuncture at GV26 can significantly improve the pathological state of cerebral hemodynamics in MCAO rats, increase cerebral blood flow and the number of microvessels, and effectively reduce the volume of cerebral infarction. Shi et al (Shi et al., 2017) confirmed that electro‐acupuncture at GV26 promotes the establishment of collateral circulation and angiogenesis, and improves neurological function. Geng et al. (Geng et al., 2020) found that the beneficial effects of electro‐acupuncture at GV26 and PC6 on post stroke rehabilitation are critically related to the activation of the delta‐opioid receptor (DOR) and also related to the inhibition of inflammatory response through the DOR‐BDNF/TrkB pathway.

In 1989, Zea Longa invested the model of MCAO, which can recover reperfusion in low damage without the need for craniotomy, and is generally accepted as model of focal cerebral ischemia (Longa et al., 1989). This method requires to cut ECA, electrically coagulate one end, insert the nylon filament into the remaining segment near the crotch and ligate the ECA branch and pterygopalatine artery (PPA). The surgery has very high requirements on tissue isolation and artery ligation, takes a very long time and is very difficult. The following improvements were made for the model in this research: (1) Penetrate the nylon filament near the crotch from the CCA. As the nylon filament head is more prone to be inserted into blood vessel, the surgical success rate is greatly improved compared to the surgery with the nylon filament penetration from ECA; (2) Only ligature ECA root rather than the branches. The traditional method requires to ligature ECA branches and PPA, but researches have proved that ischemia effects are not affected if these arteries are not ligatured (Wang et al., 2019). (3) Insert the nylon filament from inner upward side. As PPA has relatively deep position, ligation of the artery may cause great damage to rats, while failure to ligature may cause interfusion of the nylon filament into PPA during insertion, so that we can well adjust insertion angle and insert the nylon filament from inner upward side to avoid interfusion. (4) Prepare nylon filament into arc shape, mark the front end and sites at 17, 18, and 19 mm with marking pen, and polish the front end to be round and smooth with fine sandpaper. By preparing nylon filament into arc shape and polishing the front end to be smooth, the buffer force for insertion can be increased, and risk of pierced blood vessel due to excessive force can be reduced. The marks at the front end and sites 18, 19, and 20 mm can prompt the depth for the nylon filament to avoid the puncture of blood vessel due to too deep insertion.

Following focal cerebral ischemia, neuronal death is caused by both necrosis in the core and apoptosis in the penumbra within minutes to days (Deshpande et al., 1987; Leist & Jäättelä, 2001; MacManus et al., 1993). Although multiple factors are involved, disturbance of the neuronal ionic homeostasis is likely to be fundamental for these processes (Liao et al., 2010). As an advanced technology for recording the electrophysiological activities of ion channels, patch‐clamp technology provides an advanced research means for further exploring the transmembrane signal transduction mechanism of biological information. Applying the electrophysiological techniques to study the brain protective mechanisms of acupuncture at the molecular level, neurons must be obtained from crucial brain tissue using cell separation and cultivation. At present, studies on the protective effect of acupuncture on neurons in ischemic brain tissue in vitro is not common, the reason may be related to the difficulty in isolation and culture of neurons in adult rat brain tissue, and the difficulty for neurons to survive in vitro. Through previous research, our research group has accumulated experience and has completed the report on the regulation of acupuncture on the K_ATP_ channel of hippocampal neurons in cerebral ischemia‐reperfusion injury (Han et al., 2018).

Large‐conductance Ca^2+^‐activated potassium channels (BKCa) are broadly distributed in the brain, with especially high levels in the hippocampal neurons (Knaus et al., 1996). It mediates the generation of action potential repolarization and fast after hyperpolarization potential (fAHP), and plays an important role in regulating the excitability of neurons in resting state (Lancaster & Nicoll, 1987; Wann & Richards, 1994). Recurrent spreading depolarizations occur in the cerebral cortex from minutes up to weeks following acute brain injury. BKCa channel is thought to be activated during an action potential by membrane depolarization, together with a rise in the intracellular Ca^2+^ concentration. Meanwhile, due to the excessive activation of BKCa channel, the potassium efflux induced by BKCa channel are the important pathological mechanisms causing neuronal apoptosis (Huang et al., 2001). Transfection of the BKCa channel α subunit into Chinese hamster ovary (CHO‐K1) cells, which do not express endogenous K^+^ channels, or into neurons will induce cell apoptosis. Similarly, specific BKCa channel blockers also showed neuroprotection in neurons subjected to oxygen‐glucose deprivation/reoxygenation or animals subjected to forebrain ischemia–reperfusion (Chen et al., 2013).

In our previous study, we found that in cerebral ischemia/reperfusion injury rats, ischemic changes in the cerebral cortex were mitigated after electroacupuncture. Moreover, BKCa channel protein and mRNA expression were reduced in the cerebral cortex and neurological function noticeably improved (Wang et al., 2016). The present study is an extension of previous studies, aiming to discuss the effect of acupuncture on BKCa channel opening after cerebral ischemia‐reperfusion from the perspective of cell electrophysiology. We found that after cerebral ischemia‐reperfusion, the neurological function scores decreased significantly, indicating that the neurological function was obviously impaired, while the BKCa channel conductance, the open dwell time and the open probability of hippocampal CA1 pyramidal neurons increased significantly, indicating that the BKCa channel opening activity increased significantly after cerebral ischemia‐reperfusion. It is inferred that the excessive activation of BKCa channel may be an important inducement of neuronal damage. However, acupuncture at GV26 can significantly improve the neurological function score of MCAO/R rats, reduce the conductance, open probability, and open dwell time of BKCa channel, indicating that acupuncture at GV26 may play a protective role in brain by improving the excitability of neurons in MCAO/R rats. In addition, acupuncture at GV26 has a better effect on improving the neurological function score and decreasing the conductance and open probability of BKCa channel than acupuncture at non‐acupuncture point, indicating the specific therapeutic effect of acupuncture at GV26 in cerebral ischemia‐reperfusion injury.

The present study focused on the effect of acupuncture on the electrophysiological characteristics of BKCa channel of pyramidal neurons in hippocampal CA1 region 3 days after cerebral ischemia‐reperfusion in rats, but did not involve the dynamic changes of the effect on the electrophysiological characteristics of BKCa channel at different time points after cerebral ischemia‐reperfusion. In addition, this study only investigated the regulation of acupuncture on BKCa channels, and other voltage dependent ion channels (such as calcium channels and sodium channels) which were closely related to cerebral ischemia‐reperfusion were not involved. Furthermore, cerebral ischemia‐reperfusion model was chosen as the research object in this study, but whether acupuncture has the same brain protective effect on permanent cerebral ischemia model is still unclear. These work will become the main content of further research.

## AUTHOR CONTRIBUTIONS

Lin Han and Yan Shen designed the research study. Yong Wang and Yanan Zhang performed the patch‐clamp recording. Lin Han and Guanran Wang were responsible for MCAO/R surgery and acupuncture treatment. Haiping Lin and Yingying Chen were responsible for neurological function evaluation and data analysis. Lin Han wrote the manuscript. Yan Shen was the project leader and research director. All authors contributed to editorial changes in the manuscript. All authors read and approved the final manuscript.

## CONFLICT OF INTERESTS

The authors declare that there is no conflict of interests regarding the publication of this paper.

## FUNDING

This research was supported by grants from the National Natural Science Foundation (NNSF 81173339, NNSF 81603688), Tianjin Natural Science Foundation (18JCYBJC92300, 18JCZDJC99200), and Program of Tianjin Science and Technology Project (18PTLCSY00040).
